# AI-Powered Blockchain Technology for Public Health: A Contemporary Review, Open Challenges, and Future Research Directions

**DOI:** 10.3390/healthcare11010081

**Published:** 2022-12-27

**Authors:** Ritik Kumar, Arjunaditya, Divyangi Singh, Kathiravan Srinivasan, Yuh-Chung Hu

**Affiliations:** 1 School of Computer Science and Engineering, Vellore Institute of Technology, Vellore 632014, India; 2 Department of Mechanical and Electromechanical Engineering, National ILan University, Yilan 26047, Taiwan

**Keywords:** artificial intelligence, blockchain, public health, secure healthcare, smart health applications

## Abstract

Blockchain technology has been growing at a substantial growth rate over the last decade. Introduced as the backbone of cryptocurrencies such as Bitcoin, it soon found its application in other fields because of its security and privacy features. Blockchain has been used in the healthcare industry for several purposes including secure data logging, transactions, and maintenance using smart contracts. Great work has been carried out to make blockchain smart, with the integration of Artificial Intelligence (AI) to combine the best features of the two technologies. This review incorporates the conceptual and functional aspects of the individual technologies and innovations in the domains of blockchain and artificial intelligence and lays down a strong foundational understanding of the domains individually and also rigorously discusses the various ways AI has been used along with blockchain to power the healthcare industry including areas of great importance such as electronic health record (EHR) management, distant-patient monitoring and telemedicine, genomics, drug research, and testing, specialized imaging and outbreak prediction. It compiles various algorithms from supervised and unsupervised machine learning problems along with deep learning algorithms such as convolutional/recurrent neural networks and numerous platforms currently being used in AI-powered blockchain systems and discusses their applications. The review also presents the challenges still faced by these systems which they inherit from the AI and blockchain algorithms used at the core of them and the scope of future work.

## 1. Introduction

**Figure 1:**
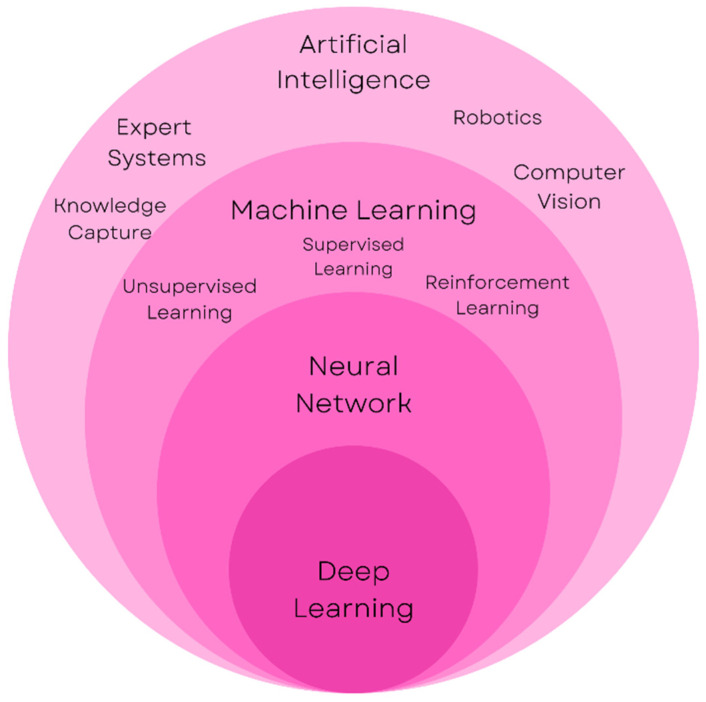
Artificial Intelligence—Nomenclature.

Modern technology is driven by Machine Learning (ML) and Artificial Intelligence (AI) based systems. Intensive research and study are being carried out in various domains of AI to integrate them in one way or another in almost all fields of computer applications. The Healthcare sector has also seen rapid growth and technological advancement, especially over the last decade. Numerous attempts have already been made to integrate AI in this sector to ease the lives of both patients and doctors. These applications are AI for drug discovery, diagnosis, intelligent clinical trials, model sharing, and patient care, including maternal care and healthcare robotics [1]. AI is a broad term that umbrellas many other models and methods that can make a system artificially intelligent. Figure 1 illustrates the nomenclature related to artificial intelligence.

Figure 1 presents a general nomenclature of Artificial Intelligence. The term AI is used for any system that uses a knowledge base in order to make a decision. Machine Learning (ML) is a subset of AI in which a machine is made to learn about decision-making based on a large set of data. A neural network is a subset of machine learning and is inspired by the human brain. It contains nodes that make up an input layer, several hidden layers, and an output layer. Deep learning is based on neural networks which use these various layers of the network to progressively extract a better set of features from a data set.

**Figure 2:**
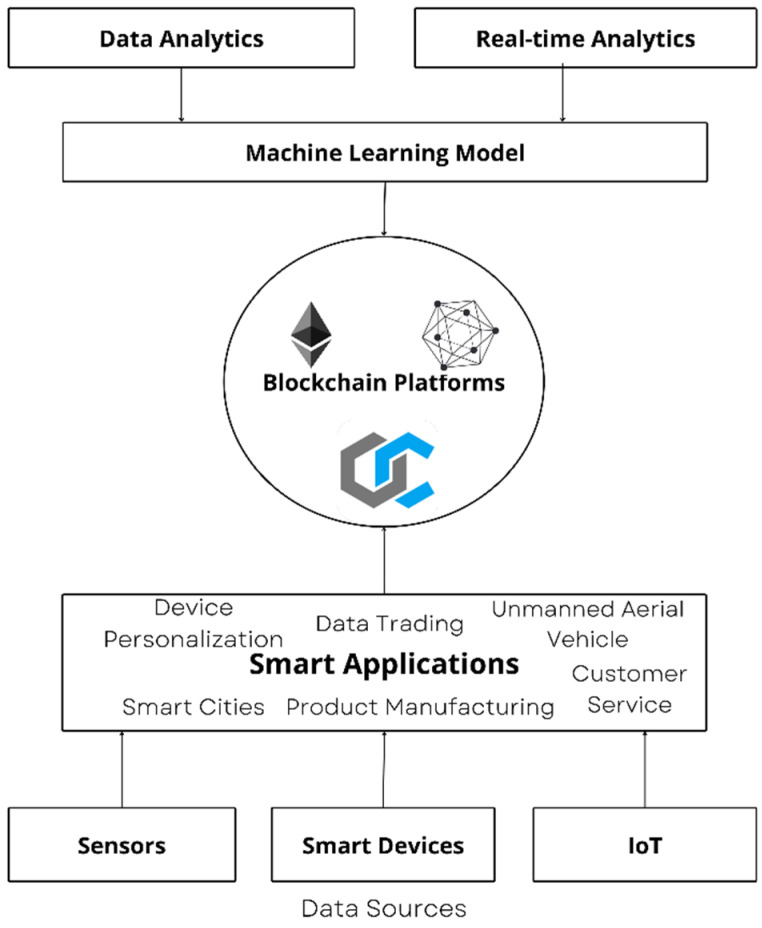
AI and blockchain relationship.

While AI has been a booming and blooming field in healthcare for a few years now, the recent advancements in blockchain-related systems have opened up new doors for decentralized environments. As the COVID-19 outbreak unfolded, people realized the importance of real-time information. The traditional patient-hospital-based healthcare system is falling out of place and the adoption of mobile healthcare has never been faster, especially with the introduction of technologies such as the Internet of Medical Things (IoMT), where smart devices can be used to track and monitor various health conditions remotely [2]. While this eases many tasks and improves reliability, a large chunk of medical data is generated and transmitted, which needs to be secured [3]. Figure 2 portrays the relationship between AI and Blockchain technology where data are collected via smart devices and sensors and sent to the smart applications which use blockchain platforms and machine learning models for analysis and prediction.

Blockchain, originally developed to support the cryptocurrency ecosystem, has recently been used in various other fields to achieve extraordinary levels of security [4]. Similarly, the healthcare sector has started integrating blockchain into various aspects of this digital age. Its features such as micro-transactions, decentralized exchanges, consensus mechanisms, and smart contracts allow for securing the privacy of the health data of patients who are key stakeholders in the healthcare domain. These data include patient clinical and trial results, billing information, medical reports, etc. [5]. Table 1 shows the list of abbreviations used along with their full form.

### 1.1. Contribution of This Review

This survey reviews the work of 120 papers taken from reputed journals and publications. Research and review papers based on blockchain, AI, and healthcare technologies were referenced for this survey. Papers on sub-domains of AI, such as those related to machine learning and deep learning and their applications in healthcare, were also considered. This survey also reviews documents discussing the integration of AI and blockchain-based systems. This survey thoroughly reviews the contributions of AI and blockchain towards the healthcare sector and what possibilities their conjunction has to offer in the near future. This survey has a primary focus to allow readers to understand what methodologies and algorithms related to AI and blockchain are used in the healthcare domain.

It presents a structured representation of how artificial intelligence could help healthcare professionals in the diagnosis of diseases such as COVID and MERS through the latest algorithms and architectures, in handling Electronic Health Records (EHRs) smartly over computer networks aiding in the research of high-quality, and in monitoring subjects in drug testing and clinical trials to evaluate how a particular drug is affecting humans. It also shows that blockchain could be used to keep a record of the origin and usage of EHRs and helps in keeping the privacy of the clients whose data are being used for medical research. It also extensively discusses the existing challenges of AI and blockchain technologies such as issues with the security of blockchain ledgers, and storage capacities and how creating standard protocols still remains majorly unexplored.

A brief comparison with existing surveys and review works is given in Table 2.

### 1.2. Survey Methodology

This survey is based on more than 100 research and review works. The work utilized the Preferred Reporting Items for Systematic Reviews and Meta-Analyses (PRISMA) methodology to select the papers used in this survey systematically. The following sections provide the details about the paper selection process.

#### 1.2.1. Search Strategy and Literature Sources

The paper aims to explain the usage of both AI- and Blockchain-based systems in the healthcare sector. It also tries to explain how the two can be merged to achieve better applications and results. The review includes all the papers closely related to the usage of AI, Machine Learning, Deep Learning, and Blockchain in the healthcare domain.

To find the relevant papers, databases such as IEEE Xplore, PubMed, ScienceDirect, Google Scholar, Web of Science, Directory of Open Access Journal (DOAJ), and Researchgate were searched from January 2012 to February 2022. The keywords selected to search over the database were Blockchain, Consensus Mechanism, Smart Contracts, Artificial Intelligence, Machine Learning, Deep Learning, Neural Network, Public Health, Smart Healthcare, Advanced Healthcare, etc.

#### 1.2.2. Inclusion Criteria

Articles published after January 2012 were taken into consideration for this review, and the remaining were excluded. This is because the work carried out in the blockchain domain before this period is not substantial and a wide range of applications of blockchain, including that in healthcare, started after cryptocurrencies became popular. 

The abstracts of the shortlisted papers were then evaluated, and only documents mentioning AI or machine learning in healthcare, AI-powered Blockchain, Blockchain in healthcare, or smart healthcare were considered for the review. As a result, this paper offers a systematic review of chosen research articles, recent review papers, technical notes, and other materials relating to recent breakthroughs in AI-powered blockchain for healthcare, structured systematically.

#### 1.2.3. Exclusion Criteria

Articles that were not written in English were initially rejected. This was carried out to avoid any discrepancy that might come from the authors’ lack of understanding of other languages. The duplicate papers were then discarded, as were those unrelated to the study based on their title. This review did not include any articles or papers published before 2012. This review did not include case reports, case series, letters to the editor, opinions, editorials, correspondence, short communications, etc.

#### 1.2.4. Result

**Figure 3:**
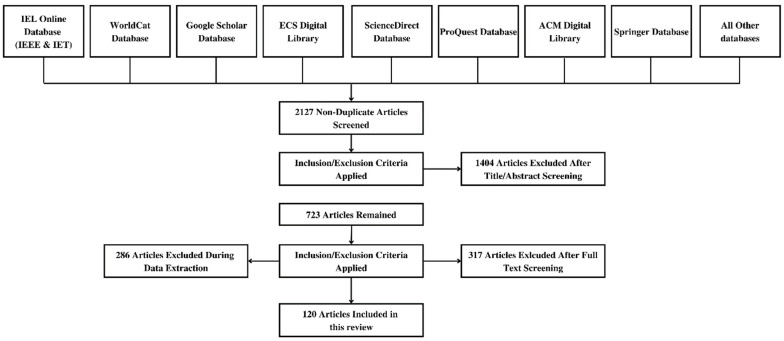
PRISMA flow diagram for the selection process of the research articles used in this review.

**Figure 4:**
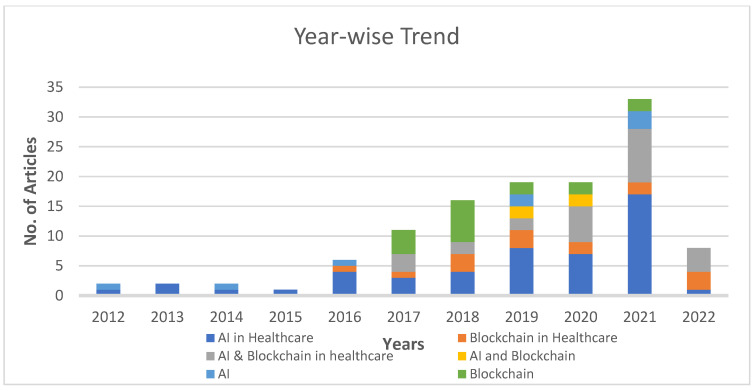
Time graph—number and year of publications studied in this review.

Initially, 2127 papers were collected for this review in the initial stage after searching based on the above-mentioned keywords. After removing duplicates and articles not in the English language, or those not found relevant based on the title, 723 papers remained. The abstract and full text were checked in some cases to analyze their relevance based on the topic of discussion, after which 120 papers were selected for the final review, as shown in Figure 3 in the form of a PRISMA flow diagram. Figure 4 also presents the year-wise time graph for the selected papers.

### 1.3. Structure of This Review

**Figure 5:**
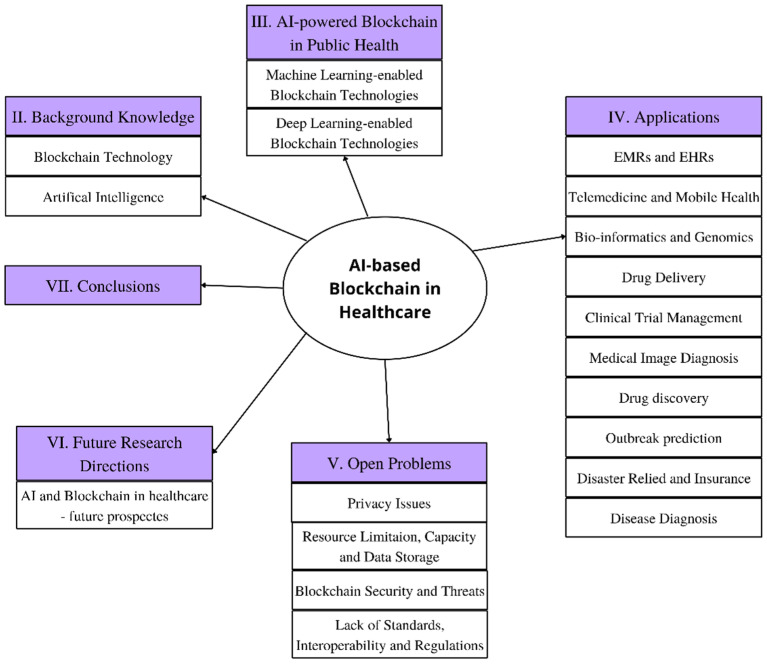
Block diagram representing the structure of this review.

The survey is organized into six sections. Section 1 covers the introduction. Section 2 lays out the foundation and basic terminologies related to Blockchain and Artificial Intelligence. Section 3 discusses the Machine Learning and Deep Learning based blockchain technologies currently used for public health. Section 4 presents the various applications of AI-based blockchain technologies in public health. Section 5 deals with the challenges in the field. Section 6 presents the scope of future research and Section 7 finally concludes the work. Figure 5 shows the block diagram representing the structure of this review.

## 2. Blockchain Technology and AI—Background

### 2.1. Blockchain

Blockchain technology is the foundation of modern cryptocurrencies, and they are called so because of their heavy use of cryptographic functions [12]. Satoshi Nakamoto first proposed the idea of using a blockchain for cryptocurrency transactions in 2008. Most of the concepts of blockchain are built around securing a transaction, primarily those involving bitcoins. It enables peer-to-peer nodes to transfer digital assets, excluding the need for any central authority or intermediaries [13]. A block, once accepted into the blockchain after the validation provided by the peers of the network, cannot be tampered with and becomes immutable. Therefore, a chain of blocks containing some data is formed, hence the name “blockchain” [14]. The blocks can record any form of transaction or agreement between two or more entities or organizations. These transactions are generally a result of any financial, industrial, or business-related activities.

In most modern applications and services, the entire system’s control lies in the hands of a centralized authority that manages and takes decisions regarding the data being handled [15]. A blockchain enables the decentralization of this control over a network. The blocks are validated using the means of cryptographic hash functions. Each block contains a hashed link to the previous valid block, and this link allows anyone to traverse back through the chain and verify a transaction in the history [16]. This also ensures the integrity of the blockchain, as changing any data in the block would also change its respective hash value, thus showing that some tampering has been carried out.

#### 2.1.1. Network Layer

To achieve its main goal of decentralization, blockchain uses a P2P network that maintains a distributed ledger that is mutually agreed upon [17]. Each system in this network is called a “node” that is responsible for carrying out the transactions. These nodes work towards finding the next valid block that can be added to the append-only ledger of the blockchain. Typically, when these transactions are related to cryptocurrencies, the nodes obtain a certain reward for finding a valid block. Each node works on top of what they think is the longest valid chain in the network.

**Figure 6:**
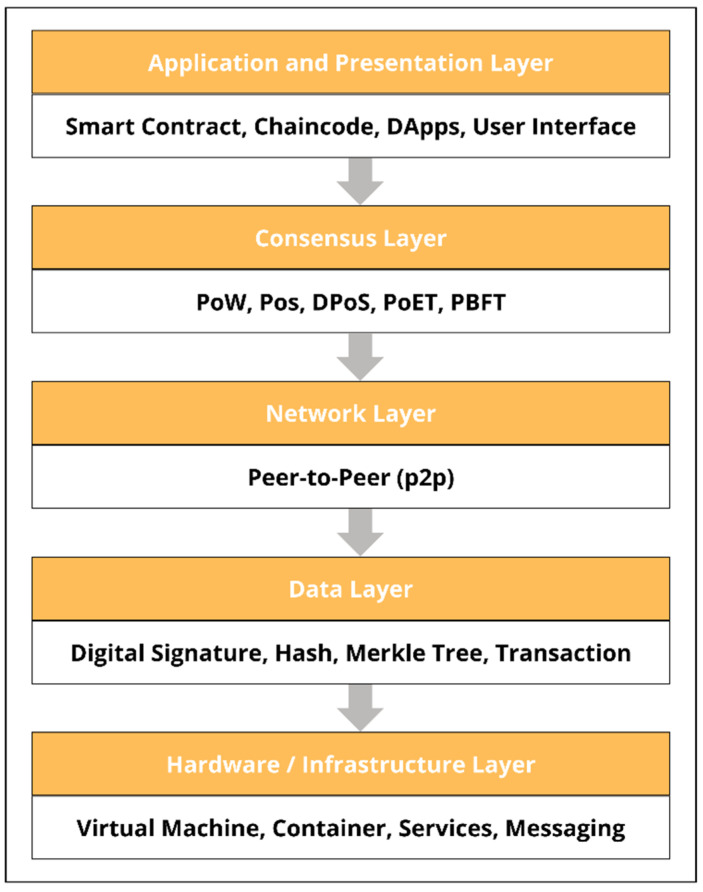
Blockchain layered structure.

The network layer ensures that the nodes can find and interact with each other. The goal behind this is such that the workload can be shared, and the control is distributed [18]. A node can enter or exit the network at any time, without affecting the working of the entire network. Figure 6 depicts the different layers present in a typical blockchain structure.

#### 2.1.2. Consensus Layer

Consensus layer is essential for the blockchain system to exist. This defines how a block gets validated in the network: the process and the requirements [19]. Consensus mechanisms have been included in blockchains as a fault-tolerant technique for transaction verification [20]. In the case of a public blockchain, the block must be verified by the participating nodes. The consensus protocol provides a method through which all the nodes in the network can agree. Nodes need to come to a mutual agreement regarding the validation of a node to ensure that only one chain is followed and that it is the truth.

Different types of consensus protocols have been proposed and all of them differ in their underlying principles and applications. Some of the mainstream consensus mechanisms that are widely used are Proof of Work (PoW), Proof of Stake (PoS), Prime Number Proof of Work (Prime Number PoW), Delayed Proof of Work (Delayed PoW), Delegated Proof of Stake (DPoS), and Proof of Activity (PoA) [21,22]. Consensus protocols focused on tolerating Byzantine faults are well received as they address blockchain [23] and are also touted for their performance. Hao et al. [19] showed that Practical Byzantine Fault Tolerance (PBFT) consistently outperformed the PoW when compared to the throughput and latency.

#### 2.1.3. Data Layer

**Figure 7:**
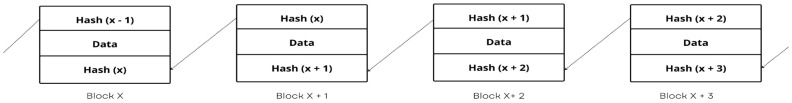
Blocks are linked together in a blockchain using a cryptographic hash. x is an arbitary block, x + 1 is a suceeding block and so on.

The Data Layer is where the blockchain network’s actual data are stored and maintained. Various cryptographic approaches are used to secure the security and integrity of the recorded transactions. The cryptographic hash of each block connects it to the previous block. In the case of Bitcoin, the hashing function is SHA256 [24], while in the case of Ethereum, it is Keccak-256 [25]. Each subsequent block in the chain strengthens the security of the preceding blocks. Figure 7 shows how hash values are used to create tamper-proof links between blocks in a blockchain network.

One block can contain multiple transaction information in a blockchain. Each block in the blockchain, along with the hash, contains a timestamp, nonce, and the root of the Merkle tree containing information about all the transactions included in that block. Merkle Tree is a tree-like data structure in which the leaf nodes contain the hash of the parent node. To know more about Merkle trees, readers can refer to [26]. The transaction data in a block can be publicly viewed by any user at any time in the blockchain. In a transaction, Externally Owned Accounts (EOAs) or contract accounts are included in the message [27]. It is in JSON format and contains information on the public and private keys created when a new EOA is created. A transaction’s creator or sender signs it with his or her private key, which can then be verified afterward.

### 2.2. The Evolution and Overview of AI-Enabled Techniques

**Figure 8:**
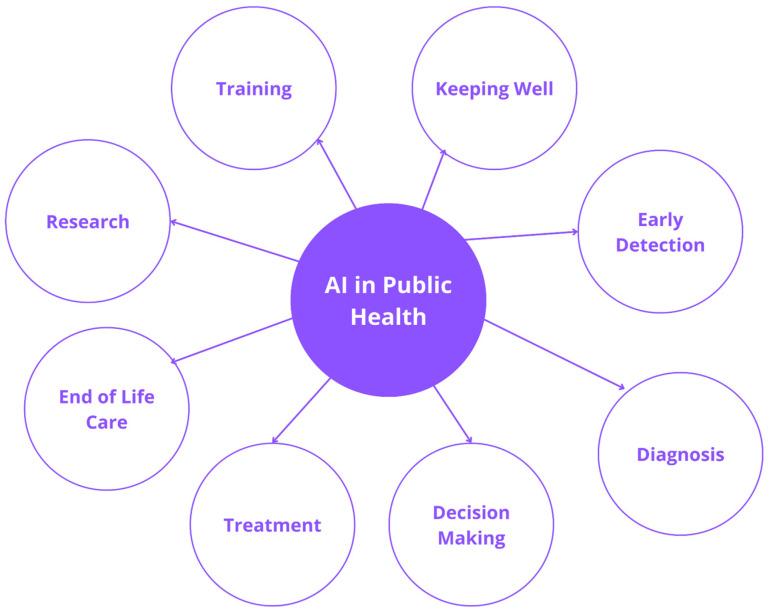
Utilities of AI in public health.

When machines perform ordinary tasks associated with human intelligence, it is referred to as Artificial Intelligence. The idea of AI was first proposed by Alan Turing in 1950. A sub-division of AI is Machine Learning (ML), where computers learn and draw conclusions from data without being explicitly programmed to do so. As advancements have been made in ML and Deep Learning (DL) systems, they have provided improved performance and unlocked new possibilities in various domains—computer vision, text and image analysis, speech processing and recognition, etc. [10]. Nowadays, many modern applications integrate ML/DL algorithms at least on some level. In addition, over the last few years, it has also started to affect the healthcare sector where various domains have already found a way to integrate AI-based systems in some form. Figure 8 illustrates the utilities of AI in public health.

ML/DL models have hugely improved healthcare epidemiology through their improved disease prediction and identification models. Tasks such as predicting the time before septic shock onset [28], body organs recognition from medical images [29], interstitial lung disease classification [30], and reconstruction of medical images [31] have become more accurate and effective with the help of ML/DL techniques. These advancements in AI-based systems have also been aided by the progress made in the fields of cloud/edge computing and big data technology. These technologies have a special effect on enabling remote healthcare services, especially for rural areas.

In the past, patients suffered from different diseases that, in the end, were mostly treated with the same drugs and the physicians could not understand why some medicines worked for some patients and not for others [32]. Now, with the advent of AI, a more personalized diagnosis can be achieved by understanding subtle disease-specific patterns from the various sources of data available.

Not only diagnosis but also procedures such as robotic surgeries for treatment are made possible using AI-based systems. Nowadays, many healthcare organizations use remote assistance services using systems such as a chatbot that takes in some questions from the patients and provides immediate responses. This is especially prominent in the mental health assistance sector. In addition, these bots can also provide medical information and answer related queries, and the underlying mechanism behind these bots is Natural Language Processing (NLP) [33].

## 3. AI-Powered Blockchain Technologies for Public Health

As advancements have been made in the field of AI and Blockchain, efforts have been made to combine the features and powers of both to create a secure and intelligent system that contains the privacy benefits of a blockchain and the predictive accuracy of AI. To improve the impact on the medical domain, adding on multiple aspects such as analytics, diagnosis, medical report validation, and decision-making, decentralized AI can be combined with blockchain [34]. 

This section illustrates the various methods and techniques that have been used thus far to combine AI and Blockchain for healthcare applications. The section is further divided into Machine Learning based methods and Deep Learning based methods which discuss some of the state-of-the-art machine and deep learning algorithms, and the blockchain platforms and types that have been used to contribute towards healthcare.

### 3.1. Machine Learning-Enabled Blockchain Technologies for Public Health

#### 3.1.1. Artificial Neural Network

**Figure 9:**
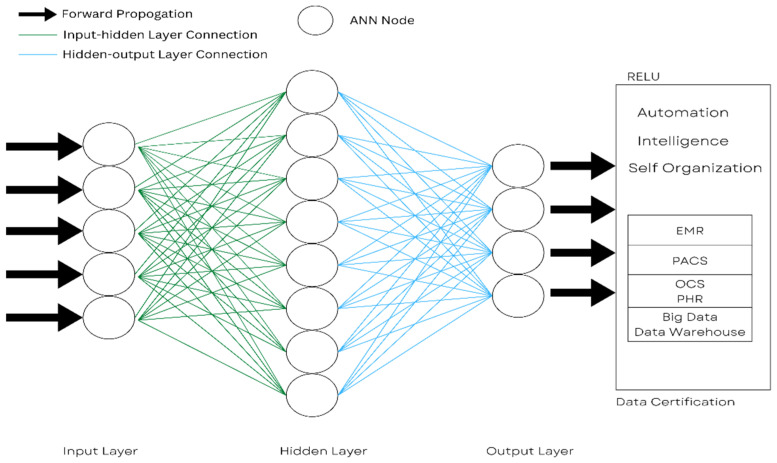
Concept of an artificial neural network for healthcare.

An artificial neural network (ANN) is a machine learning model motivated by the neural networks of living objects, especially humans. Figure 9 shows how neural networks are represented from left to right as an input, hidden, and output layer.

Blockchain technology can be applied to train neural networks, particularly in choosing the best possible configuration of weight for training or over-training. This is based on the blockchain activity paradigms and is used to overcome the time-consumption process [35]. Heavy calculations showed that such operations are possible. A neuron network blockchain [36] is also used for PHR (Personal Health Record) verification.

#### 3.1.2. Naive Bayes

Naive Bayes is a family of algorithms, based on Bayes’ theorem, wherein each feature being classified is independent of the other. It has been used to implement decentralized and collaborative AI on blockchain [37] that can be used to build a dataset and share continuously updated models over a public blockchain using smart contracts. This is to improve the efficiency and security of smart contracts by using an auto-coded intelligent contract over the Ethereum platform [38]. A study is presented [39] in which naive Bayes and random forest classifiers were trained on data that contained coronary heart disease, diabetes, and breast cancer records, and the models were trained to identify them separately using the confusion matrix as the metrics.

#### 3.1.3. Decision Tree

The decision tree algorithm belongs to the domain of supervised learning techniques. They are used for solving both classification and regression problems. It creates a set of decision rules [40] which are established with the help of training data and are used when a new set of inputs is given. Decision trees can be used effectively by physicians, especially in scenarios where decisions must be made effectively and reliably [41]. Decision trees are an excellent contender for accomplishing such jobs since they are conceptually simple decision-making models with the potential for automatic learning.

A medical diagnosis can become fairly expensive under certain conditions, and it also becomes essential to deal with the cost implications. Freitas et al. [42] has put forward a special algorithm built on top of decision trees that also includes additional costs such as testing costs, cost of delays, incorrect test costs, and risk costs. This helped in keeping the decision trees patients friendly and optimized.

#### 3.1.4. K-Nearest Neighbor (KNN)

The K-Nearest Neighbor algorithm is one of the most straightforward machine learning techniques that belong to the supervised learning category. In this method, the new input is given to the algorithm and is compared to the available cases. Based on this comparison, it is assigned the target value that occurs the maximum number of times in that group [43]. KNN has been very effective in the detection of heart diseases. By combining the genetic algorithm and the KNN algorithm [44], the attributes can be pruned based on their relevance, and the attributes that remain can be used to develop a smart model capable of predicting heart diseases with good levels of accuracy.

#### 3.1.5. K-Means Clustering

K-Means Clustering is an unsupervised learning algorithm that has the ability to group the unlabeled dataset into clusters. “K” simply fixes the number of clusters to be formed. Each cluster is associated with a centroid, and each point’s distance from the centroid should be minimized. Through multiple iterations, this distance is reduced, and each data point is associated with its most suitable cluster out of the ‘K’ clusters. 

**Figure 10:**
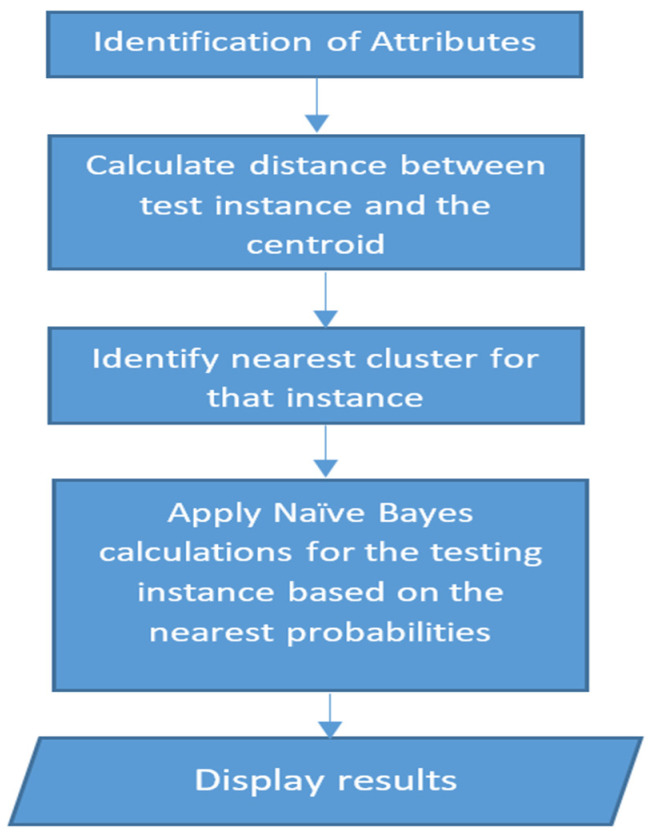
Algorithm implementing coupled K-means Clustering and Naive Bayes Algorithm.

A working model has been developed that utilized coupled integration of K-means clustering and Naive Bayes techniques where, first, the identified attributes were passed through the K-means model and were assigned a particular cluster, which in turn was analyzed by the Naive Bayes instance [45]. Figure 10 presents the flow of an algorithm implementing coupled K-means Clustering and Naive Bayes Algorithm. Another study [46] showed the use of the K-means clustering algorithm in clustering weights into a set of spline functions that were constructed using time-series data. This clustering was used to club patients suffering from chronic kidney diseases based on their spread profiles. A greedy model based on K-means clustering was used to do pattern discovery in healthcare data [47].

#### 3.1.6. Random Forest

The Random Forest Algorithm [48] is a supervised learning algorithm that is capable of dealing with both regression and classification problems. It uses the approach of ensemble learning, where multiple decision tree models are trained independently of each other, and their outputs are recorded over the input set for all the individual models. In the next step, voting is conducted, and, based on that, the final decision is taken. A method known as the Clustering based diverse random forest or CLUB-DRF has been utilized to limit the expansion of random forest models that helped in pruning the model and increasing its output rate for healthcare analytics [49].

Another important aspect of healthcare management is tracking, controlling, and limiting the spread of infections in intensive care units. Garcia et al. [50] discuss how random forest classifiers can be useful in addressing this issue by demonstrating the strength of ensemble learning. The algorithm also helps in dealing with the imbalanced dataset for this problem.

#### 3.1.7. Support Vector Machine (SVM)

The Support Vector Machine algorithm [51] is also an example of supervised learning that can deal with both regression and classification problems. The concept on which SVMs work is to create boundaries in the multidimensional space of the input data so that the data points that share similarities can be separated from other points, thus creating several segments. It uses extreme case data points to fulfill this approach. The boundary thus generated in this process is also called a hyperplane.

The use of rough set theory and support vector machines in creating an improved technique that helps deal with the redundancy of the dataset is proposed [52]. The experimental results showcased an improved accuracy of 96.56%, which was about 3.4% higher than the results received from the standalone SVM model. 

Table 3 shows a summary of works on Machine Learning-Enabled Blockchain Technologies for Public Health.

### 3.2. Deep Learning-Enabled Blockchain Technologies for Public Health

Deep learning has received a lot of attention in the last few years because it has a lot of promise in terms of making good decisions. Today’s deep learning architecture is built mainly on centralized servers, which is inefficient in terms of operational transparency, threat prevention, and trustworthiness, and is heavily reliant on statistical analysis. Deep learning technology is extensively used today in several industrial applications. Blockchain technology and deep learning, when put together, can provide massive leaps in the trustability and accountability of the systems in which they are being incorporated. Deep neural networks are provided with large volumes of information with a range of cases, which they use to learn features and generate probability vectors as an outcome. Even though deep learning models perform amazingly well on raw data, standardized data still matter in scenarios where real-life problems are dealt with. The blockchain is a worldwide database that is decentralized and verifiable, allowing all nodes in the network to store and trade data.

#### 3.2.1. Recurrent Neural Networks

**Figure 11:**
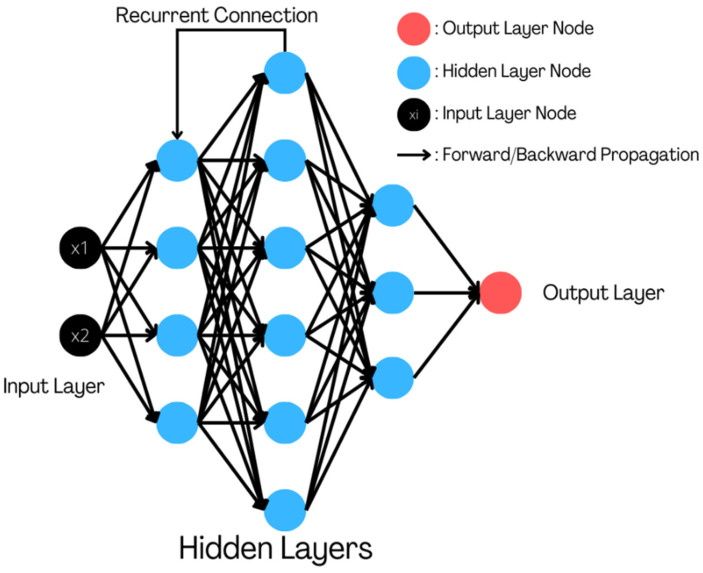
Theoretical representation of a Deep Recurrent Neural Network.

Recurrent neural networks are specialized versions of neural networks that can remember the input that is given to them. This ability possessed by the recurrent neural networks makes them very effective in problems where sequential data are being dealt with. For this reason, they are also called “hierarchical learning” [60]. Mantey et al. [61] describes the problems that institutions face while maintaining electronic health records (EHRs), such as trust issues within the organization and being vulnerable to hacking. The research suggests a storage technique employing a blockchain-Hyperledger to work with medical data with improved confidentiality and protection. Any patient’s medical records cannot be viewed without their authorization. Using RNNs along with graph neural networks (GNNs) was proposed [62] to predict the prescription of the next period. RNNs are used for keeping track of the status of the ongoing sequence of prescriptions and GNNs for keeping track of medical events. This technique uses existing electronic health records and helps to provide accurate predictions for upcoming prescriptions. Figure 11 portrays the theoretical representation of a Deep Recurrent Neural Network.

#### 3.2.2. Deep Autoencoder

A deep autoencoder is made up of two symmetrical deep-belief networks, one with 4 to 5 shallow layers that is responsible for encoding and the other with 4 or 5 shallow layers for the decoding task. They function by reducing the input into a smaller representation and recreating the output in a similar form as the input. They are deep neural networks that are used to replicate the values at the output layer with the same number of neurons in the output layer. Training datasets with a Deep Boltzmann machine rather than a Restricted Boltzmann machine yields superior results in terms of unsupervised learning tasks [63].

#### 3.2.3. Deep Belief Network

**Figure 12:**
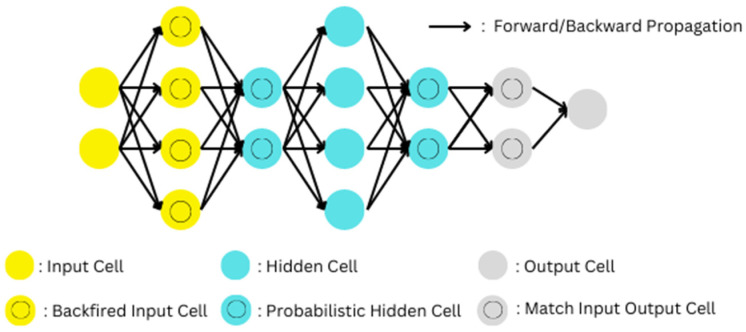
Theoretical representation of a Deep Belief Network.

A deep belief network (DBN) is a generative graphical model, a form of deep neural network, made up of many layers of hidden nodes connected by links between levels but not between nodes at the same level. Even though the DBNs have been phased out of use today, since they are generative networks, they still have a great capacity for dealing with both unsupervised and supervised learning problems. Movahedi et al. [64] discuss the latest contributions that DBNs have provided to the domain of electroencephalography. Figure 12 depicts the theoretical representation of a Deep Belief Network.

#### 3.2.4. Deep Convolutional Neural Network

Convolutional neural networks (CNN) are specialized deep neural networks that deal with image recognition and image processing. The inputs that are served to the CNNs are images. The images are usually made up of thousands of pixels that have values in several channels. The unique feature of the CNN architecture is the utilization of convolution, which is an image processing technique that helps in reducing the weight count to a manageable number, which results in faster processing of the images. Yadav et al. [65] have discussed the use of the VGG16-based CNN model for identifying the presence and spread of pneumonia in digitized X-ray scans.

**Figure 13:**
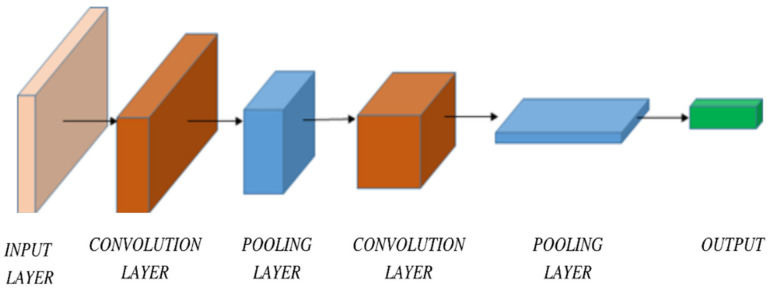
Basic structure of a Deep Convolutional Neural Network.

The COVID-19 outbreak has affected the lives of people throughout the world. Gaur et al. [66] demonstrate the use of a transfer learning-based CNN model in detecting the COVID-19-affected lung scans and distinguishing them from viral pneumonia-affected lungs and normal lungs. The model was trained on the publicly available dataset and tested on metrics such as accuracy, specificity, and F1 score. The model achieved a total accuracy of 92.93% and proved to be a helpful computer vision setup. Figure 13 shows the basic Structure of a Deep Convolutional Neural Network, containing one input and output layer and two convolutions and pooling layers in between them.

#### 3.2.5. Deep Generative Models (DGM)

A Generative Model is a form of unsupervised learning approach that aids in the comprehension of many sorts of data spreads, and it has acquired a lot of attention in recent years. To produce new points with some variability, all generative models attempt to learn the true data distribution of the training set. DGMs are capable of approximating multidimensional probability distributions [67]. Generative adversarial networks (GANs) and their capabilities are evaluated in creating more images that can be utilized to train deep learning models and how effectively the GANs solve the problem of insufficiency of data [68].

#### 3.2.6. Deep Reinforcement Learning (DRL)

DRL, or deep reinforcement learning, is a rapidly growing field that combines reinforcement learning and deep learning. It is one of the most extensively utilized and popular machine learning algorithms as it can handle a wide range of previously unattainable complicated decision-making tasks. Table 4 represents a summary of works on deep learning-enabled Blockchain Technologies for Public Health.

## 4. Applications—AI-Powered Blockchain Technologies for Public Health

AI-powered blockchain can have a huge number of applications in healthcare. As a shift is happening toward the usage of smart devices for patient health monitoring and tracking, a large amount of data is produced which can be leveraged by various AI-based systems for decision-making [74]. As healthcare has advanced, nowadays physicians can diagnose and treat many more diseases than they could before. However, an efficient and reliable diagnosis is still an issue in many cases. This is where AI-based systems can provide reliable support for performing a diagnosis and providing the best course of treatment.

Similarly, Blockchain also provides various ways to improve the healthcare system. It finds its application in patient data management, pharmaceutical research [75], managing Electronic Health Records (EHRs) [76], decentralized apps (dapps) for healthcare [77], enhancing the security of data [3], etc. A parallel healthcare system based on blockchain was proposed to improve the accuracy of diagnosis and also make the treatment more effective [78].

While both Blockchain and AI have helped the healthcare domain in more than one way, they still have some issues when used on their own [79,80,81,82,83]. As the idea of decentralized AI came into existence [84], several limitations have been overcome with its help. Krittanawong et al. discussed the integration of blockchain with AI for cardiovascular medicine and also summarized applications that can be realized by combining decentralized blockchain platforms for data security and AI computing, for data analytics [85].

### 4.1. Electronic Medical Records and Electronic Health Records

Electronic Health Records (EHRs) are longitudinal data collected in electronic format during the routine delivery of healthcare. Maintaining EHR provides a lot of insight and opportunity to enhance patient care, improve performance measures in clinical trials and enhance the identification of probable diseases in a population in the near future. It also helps in analyzing whether new research and discoveries in healthcare are providing any improved outcomes or not.

EHRs need to be kept secure as they can contain any personally identifiable information (PII) of the patient that, if exposed, can lead to a breach of data privacy [81]. Many of the EHR systems, however, face security, integrity, privacy and management challenges [86]. Using blockchain technology to manage and maintain EHR can help remediate this issue. Shahnaz et al. put forth a framework that used smart contracts to maintain patient records [87]. Using Ethereum as the blockchain network, the system had an administrator and user, where a role was assigned to the user by the administrator, which can be either a doctor or a patient. Functions such as adding patient data, viewing data, and deleting data were provided, and the data were directly added to the blockchain network, thus ensuring security. Similarly, Vora et al. proposed Bheem: a blockchain-based system to manage EHRs [88]. The system contained patient nodes, provider networks, and proxy nodes. The goal here was to provide secure and easy access to data to patients, providers, and third parties.

Another important contribution that blockchain makes towards maintaining EHRs is interoperability [89]. Patients’ lifetime data are generally scattered across multiple institutions as life events take them from one organization to another. Having the data stored in a single, secured network that can be accessed by multiple providers may help address this problem. Zheng et. al. [90] proposed a design for sharing medical data using blockchain and cloud technology. The design also included a machine learning based data-quality inspection module.

**Figure 14:**
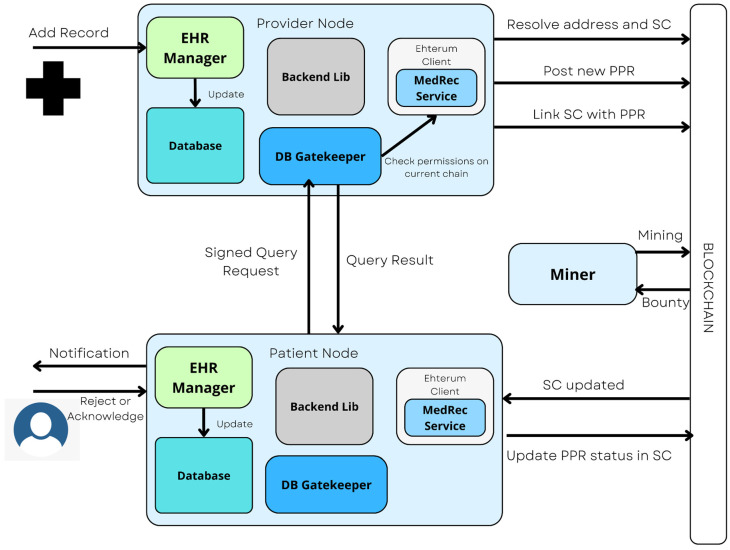
Illustration of a system where a provider adds an EHR for new patients using blockchain.

EHRs are also very useful when used with AI systems. Building upon traditional Early Warning Systems (EWS), which aim to predict acute critical illness, data from EHRs play a crucial role in improving the performance and accuracy of predicting acute critical illness [53]. Methods such as ML and NLP are also applied to EHRs to obtain quantitative data from various tests in the form of visual synopsis [76,91]. Figure 14 shows an illustration of a system for adding EHR for a new patient using blockchain in which a record is added by a provider node and acknowledged by the patient using a signed query request. 

AI can be used to analyze medical records and offer information to physicians. Based on past data and family history, algorithms can use EHR to forecast the possibility of a disease. To train AI systems, large volumes of data are used, and the algorithm develops a set of rules that relates its observations to the final diagnosis along the process. When AI is provided with fresh patient data in the future, it may make a judgment about the patient based on prior experience [92]. AI techniques like text-recognition can be used to generate EHRs form hand-written documents and saved on the blockchain [93].

Customers have easy access to copies of their electronic medical records, which may then be licensed to other medical institutions or third-party platforms. The doctor must upload and adjust permissions based on client consent because the customer has read-only access. Doctor management contributes to the client-centered paradigm of the system. Blockchain technology can allow for the interoperability of patient and medical staff identification assurance and verification. A verification platform has been created using blockchain that verifies physicians’ identities and certifications regularly, ensuring patient safety and high-quality medical treatment [94].

### 4.2. Telemedicine, Mobile Health, Remote Patient Monitoring, and Personalized Medicine

Telemedicine and remote patient monitoring have been the need of the hour since the COVID-19 pandemic hit. Most of the current telehealth and telemedicine systems are centralized and lack privacy, security, and openness [75]. The connection between the patient and the medical personnel is made through a public cloud service, which adds to the security risk. This is especially true when a patient from a remote location seeks medical assistance from a doctor who is located far away.

Blockchain can help improve the telehealth system by offering remote health services in a decentralized manner which will make the data tamper-proof and provide the necessary transparency [95]. Mobile health activities can be performed in virtual environments in cases where the patient needs to be kept isolated, such as in the case of a COVID-19 infection [96]. Subramanian et. al. [97] proposed a design for a blockchain-based diabetes consortium that can help prioritize diabetes patients during the time of a pandemic and provide remote healthcare.

Personalized predictive modeling, which focuses on constructing tailored models for specific patients, has proven to be more effective in leveraging a wide range of health data than global models trained on the entire population. Individual features are extracted using data from similar patient groups in personalized prediction models. The time fusion CNN, unlike a regular CNN, can learn both local temporal correlations and contributions from each period. To rank comparable patients, the probability distribution developed as part of the similarity learning process is employed [98].

**Figure 15:**
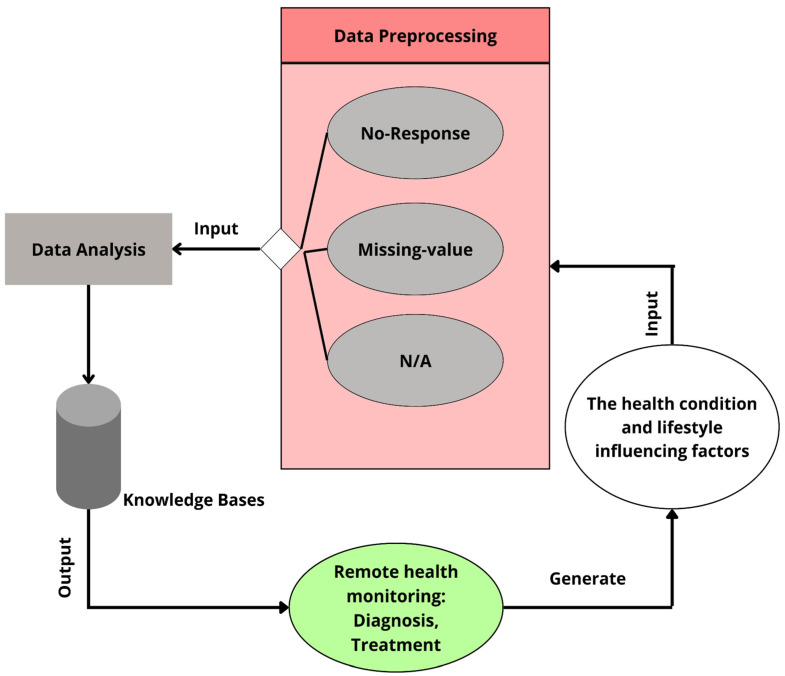
Data processing model of EHRs for remote monitoring.

CNN models can also help in finding frequently related health factors that may influence health. Patients’ medical data are first collected and then preprocessed. To discover relationships between disorders such as obesity, high blood pressure, and diabetes, the most important indicators are picked from the analyzed data. Then, the productive correlation among the components is examined to see which factors are positively and negatively connected. Finally, the needed information is derived by selecting the regular co-occurring factors [99]. Figure 15 shows the Data Processing model of EHRs for remote monitoring where the data is gathered from remote health monitoring devices, pre-processed, analyzed, and then used by the knowledge base to derive and output.

### 4.3. Bioinformatics and Genomics

Significant work has been carried out in the domain of Bioinformatics under Artificial Intelligence and Machine Learning. To aid in the prediction of sequence requirements of DNA-protein binding, a high-order convolutional neural network (HOCNN) was devised, which used a high-order encoding strategy to incorporate high-order relationships among nucleotides [100]. It also uses a multi-scale convolutional layer that helps in capturing the motif features of different lengths. Li et al. proposed Automated ICD-9 Coding using a deep learning approach to overcome the cost, time consumption, and inefficiency of manual coding [101]. The DeepLabeler framework assigns ICD-9 codes automatically by combining CNN with the Document to Vector technique. 

To overcome one of the difficult tasks in sequence analysis, which is to identify protein remote homology, a Long-Short Term Memory (LSTM) based predictor was proposed in [102]. The DL-based technique captured profile-based patterns from the Polysaccharide storage myopathy (PSSMs) and the method successfully outperformed other methods in this area.

### 4.4. Drug Delivery and Pharmaceutics

Targeted drug delivery has seen numerous advancements over the last decade, including the emergence of implantable microchips for the controlled delivery of drugs [103]. These systems help in achieving maximal efficiency with minimal side effects. The performance of these systems, however, can be further improved by using AI and ANNs. AI-based platforms can analyze biological data to identify potential targets for drug delivery and search from a variety of pharmaceuticals that can result in a better and more efficient drug delivery process [104]. 

He et al. [105] listed five reasons why ML is best suited for drug delivery, especially for infectious diseases, which include the ability to find features in a complex and large-scale database, the ability to form new rules and identify unforeseen patterns, great data processing and analyzing speed, possibility of operating at the point of care via embedded software on mobile and learning ability from unidentified and new microorganisms.

Along with ML techniques for accurate and efficient drug delivery, pharmaceutical companies can leverage blockchain to manage their supply chain and ensure drug safety. Abbas et al. [55] proposed a system consisting of two modules: a machine learning system using the LightGBM and N-gram models to recommend the accurate drug to the customer, and a blockchain-based supply chain management system, capable of monitoring and tracking the process of drug delivery by the company continuously. Jamil et al. [106] also proposed a similar system, focusing on secure drug supply chain management from smart hospitals. A framework was presented to optimize the drug distribution process to ensure the equilibrium between supply and demand [107]. The framework required the drug regulatory authority to authenticate users on a blockchain and to monitor the drug delivery process. Hyperledger can be used to fight counterfeit drugs in the pharmaceutical industry [108].

### 4.5. Clinical Trials Management

Clinical trial management plays a critical role in developing and validating medicines and drugs for their usage and consumption in the whole world. Clinical trials are carried out in two phases: patient selection and patient monitoring. For patients who desire to engage in a clinical trial, there are certain eligibility, appropriateness, motivation, and empowerment requirements. The medical history of a patient may preclude them from seeking treatment. Patients who are eligible for testing may not be in the proper stage of disease or belong to a certain sub-phenotype targeted by the drug being tested. Blockchain offers a damage resistant and decentralized data storage for storing medical information of the patients [109]. 

Unsupervised machine learning can be used to uncover patterns in clinical variables that can be used to identify patient phenotypes that will benefit from the intended therapy or intervention. Because unstructured data are so vital for phenotyping and forming representative cohorts, incorporating more patient data is a crucial step toward generating good, representative cohorts [110]. 

Using DL algorithms such as NLP and Optical Character Recognition can help in identifying patient cohorts. AI and machine learning technology may also be used to predict a patient’s risk of dropping out dynamically or to detect the onset of patient behavior that could suggest a problem in a phased manner [111]. Blockchain tasks in trial management can be automated with multiagent using reinforcement learning [112].

### 4.6. Medical Imaging Diagnosis

A comprehensive overview of techniques and uses of CNNs and their variants in medical image interpretation, including the detection of the most recent global pandemic, is important. The authors leverage their own and others’ experiences with CNN applications to provide insight into many state-of-the-art CNN models, issues in building CNN mode, and motivation for medical image comprehension academics and practitioners to employ CNN more broadly [113].

AI can spot indicators of disease more precisely and quickly with the use of medical imagery such as CT scans, MRIs, X-rays, and ultrasounds. It benefits patients by allowing a more accurate diagnosis of illness and more precise therapy options. Deep learning has greatly improved the detection of skin cancers and legions by applying CNN architecture for identification [92].

In an automated approach for micro-organ segmentation, there are two processes: localization and segmentation, which are especially important when there is little training data. The localization stage extracts the region of interest after projecting photos to a common space using a graph-based multi-group image registration technique. In the segmentation stage, a voxel-wise label map is acquired using a trained convolutional neural network. Deep learning is widely used to perform sequential and independent cell identification and classification.

A single architecture is used to identify the locations of cells and classify the detected cells concurrently in the proposed synchronized deep auto-encoder network for simultaneous detection and identification of cells in bone marrow histology photographs [114].

### 4.7. Drug Discovery and Manufacturing

The application of deep learning also extends to the domain of drug discovery and manufacturing. Using fully integrated DNNs to develop models is a simple way forward when compounds are presented with a uniform amount of cell definitions [115]. A huge number of 2D topological descriptions are used in DNN research in the Merck Kaggle challenge database, and DNN has shown a slightly higher 13 target performance out of a total of 15 than the traditional RF approach. It also includes a paper claiming that their DNN models for multiple jobs were victorious in Tox21. 

### 4.8. Outbreak Prediction

The recent COVID-19 break has presented itself as a big challenge to our balanced world and societies. In situations such as these, deep learning techniques can prove to be extremely useful and relatively quick. Marzouk et al. [116] showed a work that forecasts the occurrence of the COVID-19 epidemic in Egypt. To achieve the main purpose, the following actions are taken: acquiring, preparing, and pre-processing COVID-19 data prior to integrating it into neural networks, splitting datasets into training and testing sets, applying deep and machine learning models, evaluating the performance of the model using evaluation metrics, and forecasting with the most suited model LSTMs: a sort of deep RNN known for its internal memory system. The proposed model has the ability to process large volumes of information and identify dependencies. This network, which is made up of many components, employs a chained architecture. Along with that, the authors have also utilized CNNs for spatial knowledge gathering and Multi-Layered Perceptron Neural Networks that are extremely useful in modeling complex datasets, especially diseases.

### 4.9. Disaster Relief and Insurance

Medical insurance is a crucial agreement for any patient, the integrity of which needs to be maintained properly. In a typical insurance system, three parties are involved: the patient, the hospital, and the insurance company. Managing the expenditure records can sometimes become difficult, and it also lacks transparency. Insurance is also prone to fraud, which can be very hard to track. Blockchain and AI can help mitigate these risks.

**Figure 16:**
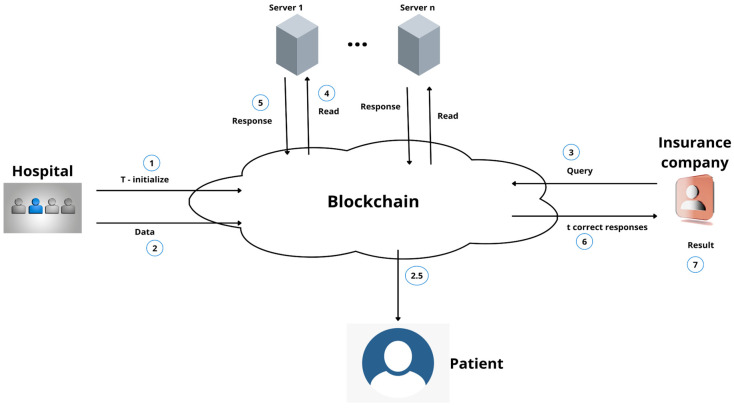
The basic structure of MIStore. Arrows represent the flow of data while the numbers represent the sequence of the processes.

Yuliang et al. proposed a fraud-resilient medical insurance claim system based on HFDA [117]. Zhou et al. proposed an insurance record storage system based on blockchain, called MIStore, to provide high credibility to users along with decentralization and efficient verification as all parties can view the agreements and transactions happening as they are logged in the blockchain [109]. Not only does this help in mutual agreement and understanding from all the stakeholders, but it can also help in reducing the cost of medical care through better insurance claim coordination. Figure 16 shows the basic structure of MIStore proposed in [109] that provides a smooth insurance claim system using blockchain that involves all the required stakeholders.

### 4.10. Disease Diagnosis

Deep learning is a data-modeling approach that uses many processing layers to reflect a high level of abstraction. DL approaches have progressed to the point that they are now routinely employed by researchers all around the world to identify a variety of illnesses. DL has shown exceptional performance in a number of applications by recognizing long-range correlations and effectively creating dense hierarchical order features. Convolutional Neural Networks (CNN), Recurrent Neural Networks (RNN), and other deep neural networks are used in deep learning (RNN).

A study was presented that evaluated a CNN-based approach for predicting diabetic retinopathy prognosis [118]. They added the BN layer to the classic LeNet model, by which this CNN model was reorganized to create a new BNCNN model. The suggested technique obtained state-of-the-art training and testing accuracy of 99.85% and 97.56 percent, respectively. The BNCNN model proved efficient in reducing gradient diffusion and boosting the network’s training speed and efficiency. The model-based reasoning (MBR) method based on EMR and NLP for illness diagnosis achieved an accuracy of 95.86 percent.

The authors also looked at research that used pediatric EHR data to offer a way for applying an RNN-based predictive diagnostic algorithm. This work used NLP concepts to generate a feature vector for a collection of Chinese unstructured EHRs and then moved the values into a sentence vector. The suggested bidirectional Recurrent Neural Network was used to identify the patient’s symptoms and their association. A dataset of 81,476 pediatric records was utilized to train as well as test the model which had an accuracy of 80.912.

## 5. Open Problems—AI-Powered Blockchain Technologies for Public Health

**Figure 17:**
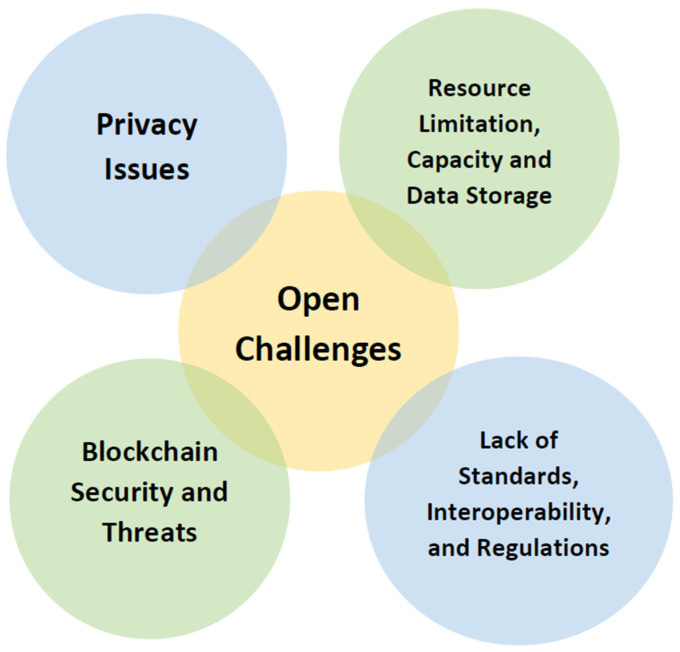
Open challenges in using AI-powered Blockchain for public health.

The use of blockchain and artificial intelligence technology in the field of healthcare has revolutionized many sectors, but all the advancements come with a cost. The new technology and implementations demand extremely high operation costs, lack of flexibility, and huge amounts of data storage on the local systems. In certain situations, due to the absence of an arbitrator, the legal threats of blockchain cannot be dealt with. Figure 17 provides an overview of the current challenges faced by AI-powered blockchain systems in public health.

### 5.1. Privacy Issues

Healthcare databases consist of personal and confidential information of the patients, and hence preserving their privacy is of utmost importance. There are quite a lot of privacy issues linked to sharing of data in public blockchains. The decentralized and transparent property of blockchain poses major threats to the data. Blockchain connects various members of an organization and, in this scenario, it can be difficult to identify the subject responsible for the illegal use of the data. The scenario can get worse when parties outside of the healthcare network are added to the blockchain. Various protocols such as distributed consensus have been introduced to overcome the privacy issues interlinked with blockchain technology [119]

### 5.2. Resource Limitation, Capacity, and Data Storage

The requirements for running a successful blockchain and the requirements for running Artificial Intelligence on a system are quite different and require a good amount of data storage and capacity in a system. Current blockchain technology also comes with a limitation on the number of transactions that can be performed per second in the network. This also comes with the fact that heavy computation power is required to handle the transactions on a scale. This also leads to high energy consumption which has been a lingering concern with blockchain technology. The process of consensus and other protocols lead to a great amount of energy consumption which can hinder the speed and efficiency of the process [119].

Along with that, the recent advancements in the field of big data analysis have increased the need for data storage since every project requires a good amount of data to be analyzed to reach the conclusion or obtain a definitive output with higher prediction power. The provision for some of the greatest big data analysis tools is required, but it would cost a lot more than usual and there will be certain drawbacks of these tools as well [96]. 

AI-based systems also run on accelerated hardware. Most of the AI architecture involves complex calculations, with many algorithms requiring processing a huge amount of data in each cycle, which causes latency. Although faster networks can improve the latency, many algorithms need to wait for a complete cycle before processing the next set of data.

### 5.3. Blockchain Security and Threats

Recent research projects have emphasized some of the inherent security weaknesses of blockchain technology. There have not been a lot of recent advancements in the blockchain protocols and hence it is easier for the attacker to analyze and determine the loopholes in the existing system. There are various threats such as data modification and stealing which are possible in the case of blockchain technology. The malicious attackers also have the provision of injecting false data or sample inputs which can not only affect the AI learning but also completely change the final output of the model, which poses a big threat to the customer’s health and wellness. Blockchain technology has also been found to be susceptible to cyber-attacks such as the DNS (Domain Name System) attack and mempool attacks [96]. 

### 5.4. Lack of Standards, Interoperability, and Regulations

Before coming up with the implementation plan for blockchain and AI applications in the healthcare sector, various regulatory laws should be referred to. Most of the integration of AI into healthcare is still limited to academic research, and there are only a few practical applications that have been possible, primarily because of interoperability issues. There also is a question as to how AI diagnostics systems should be placed in the healthcare sector. It can be either placed as an AI as a helping hand to the doctors or AI replacing the doctors. However, both of these methods come with the thought of “dangerous AI” considering the sensitivity of the healthcare domain.

One of the biggest challenges in adopting blockchain widely across healthcare sectors is the question of ownership. As private and public blockchain exists, it becomes a valid question as to who owns the data in the case of either of the two blockchain solutions. Sometimes, due to the absence of an authority that can be held responsible for various actions of the technology, there are quite a lot of legal and regulatory law issues that exist [96]. There can also be a lack of trust between the different stakeholders involved and limited open standards, which lead to a difficult exchange of information between the different health organizations. In addition, although economical in the long run, the initial setup of a blockchain-based system is costly and may not be feasible for smaller healthcare organizations.

## 6. Future Research Directions

**Figure 18:**
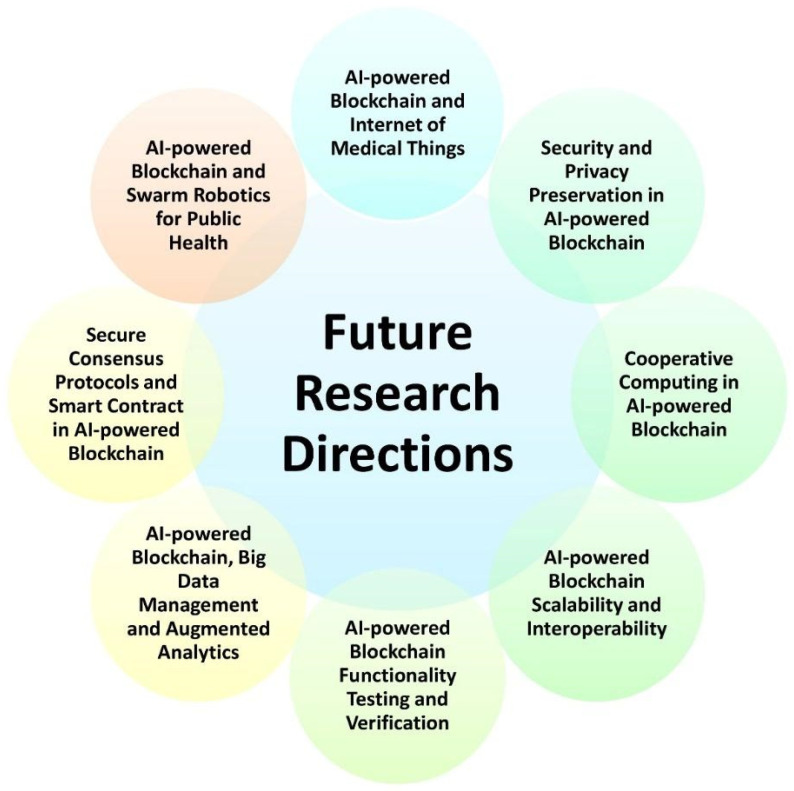
Future research directions for AI-powered Blockchain in public health.

The model consisting of deep learning methods and Blockchain technology requires more detailed research since the field is evolving at a very fast pace. Deep learning can have a great amount of application in detecting myopia and other internal body diseases and using it with blockchain technology provides both accuracy and security to the patient’s details. To collaborate in both fields, it is important to carry out intense research before submitting a design of the proposed model, and this could also come in our future research objectives. These models will also be able to detect chronic diseases such as cancer, diabetes, arthritis, and some major heart diseases with much better accuracy and maintain the security of the data [119]. Figure 18 provides an overview of the possible future research directions that can be taken for AI-powered blockchain.

There is still scope for a lot of improvement in the security and privacy of AI-powered blockchain technology. Implementing the latest hash algorithms, and using a more rigid verification system are some research areas that can be explored in the future. Using two-step and multi-step authentication for every node on the blockchain will have its advantages and disadvantages but cannot be determined for this research interest. Using the traditional consensus protocols such as proof-of-work and proof-of-stake would not be a good idea since these protocols have been in use for approximately the last 10–11 years and various loopholes have been found in them. New consensus protocols such as proof-of-elapsed-time and Delegated Proof of Stake could be applied in the AI-powered blockchain, which will improve the security and efficiency of the system to a great extent.

Cooperative computing is considered to reduce the time of computing the hash of a block when compared with a non-cooperative computing strategy [120]. Using a diverse collection of different technologies would increase the working speed of AI-powered blockchain, and integrating it with existing systems is also a future research possibility. Improving security and ensuring privacy preservation also need to be of great importance if AI-powered blockchain systems are to be commercially used in the healthcare domain.

## 7. Conclusions

The paper gives an overview of Artificial intelligence and blockchain integration and how they can be applied in the field of healthcare. Several research projects have been carried out on the application of AI and Blockchain in this field, especially during the COVID-19 pandemic. There have been various suggestions to improve the existing models, and all of these have built a future image for blockchain and AI in healthcare. The market of blockchain is expected to cross approximately $500 million by the year 2022. This paper provides a comprehensive review of 120 research articles on the topic of AI-powered blockchain applications in healthcare. It also provides a clearer picture of how artificial intelligence could improve our abilities to identify and quickly act on the diagnosis of diseases and how blockchain could structurally improve the security of medical records and preserve the privacy of the record owners. Various features of blockchain have been discussed which allow it to provide security to the EHR and other confidential medical records. Artificial Intelligence and machine learning integration usually help in automating the process of data analysis and scanning. 

The paper has given a brief overview of how the blockchain system works and the different layers present in the design. There are mainly five layers present which include the Application and Presentation Layer, Consensus Layer, Network Layer, Data Layer, and Hardware/Infrastructure Layer. Furthermore, the integration of artificial intelligence has also been the point of focus. Different machine learning algorithms such as decision trees, support vector machines, KNN, K-means clustering, and artificial neural networks have been discussed. These algorithms have been used for years for improving the process of data extraction, data mining, and data analysis. The integration of machine learning and Artificial intelligence into blockchain technology would provide an automated, secure and decentralized system for data storing and analysis of important and confidential medical records. 

The work explores some of the applications of AI-powered Blockchain in the healthcare domain such as maintaining and managing EHRs, introducing telemedicine and remote health monitoring, bioinformatics and genomics, clinical trial management, drug delivery, medical image diagnosis, drug discovery and manufacturing, outbreak prediction, disaster relief and insurance, and disease diagnosis.

There are certain limitations of the existing system despite the various advancements happening in the field of machine learning and blockchain. One of the main functionalities of the blockchain system is the level of security it provides to the data being stored on its system, but there are still various privacy issues that persist in this advanced technology. External party access to the database, decentralized system, and various other features of public blockchain can make the data insecure, and attackers can easily gain access to it. Along with these issues, there are some legal restrictions while using blockchain and artificial intelligence technology on medical databases, and these restrictions can act as a major challenge when developing AI-powered blockchain. The paper has also discussed future research possibilities which have the capability of overcoming these limitations and challenges.

## Figures and Tables

**Table 1:** List of abbreviations used along with their full form.

Acronym	Definition
AI	Artificial Intelligence
ML	Machine Learning
DL	Deep Learning
CNN	Convolutional Neural Network
ANN	Artificial Neural Network
NLP	Natural Language Processing
DRL	Deep Reinforcement Learning
RNN	Recurrent Neural Network
GNN	Graph Neural Network
KNN	K-Nearest Neighbor
DGM	Deep Generative Model
DRL	Deep Reinforcement Learning
PII	Personally Identifiable Information
EHR	Electronic Health Record
LSTM	Long-Short Term Memory
MBR	Model-Based Reasoning

**Table 2:** Comparison with other similar review articles.

Reference and Year	Number of Articles	Period	One-Phrase Summary	Blockchain	AI-Powered Techniques		Open Challenges	Future Directions
					Machine Learning	Deep Learning		
This Review	120	2012–2022	A systematic review of AI and Blockchain powered techniques in healthcare	✓	✓	✓	✓	✓
[6], 2021	208	1998–2021	Discusses security and privacy implications of applying ML/DL techniques in healthcare	✗	✓	✓	✓	✓
[7], 2021	150	2002–2021	Ethics of implementing ML in healthcare	✗	✓	✗	✓	✗
[8], 2021	68	2016–2020	Review on the usage of data from health devices in ML-based healthcare systems.	✗	✓	✓	✗	✗
[9], 2021	159	2008–2021	Integrating ML models into IoT-based healthcare devices	✗	✓	✓	✓	✗
[10], 2021	158	2014–2021	Review of smart healthcare systems including usage of wearable devices and smartphones for monitoring.	✗	✓	✓	✓	✓
[11], 2019	72	2014–2019	ML adoption for Blockchain-based systems to make them more resistant to attacks.	✓	✓	✓	✓	✗

**Table 3:** A summary of works on Machine Learning-Enabled Blockchain Technologies for Public Health.

Ref.	Healthcare Application	Security Challenges	Dataset	Machine Learning Approaches Used	Blockchain Types Used	Blockchain Platform	Key Contribution	Limitations
[53]	Personal Health Record (PHR) verification	EHRs are not used much because of privacy and security concerns	Sample medical data of patients are used.	Neural Networks	Blockchain using Hyper POR algorithm	Ethereum	Verification of medical information with accurate image data extraction and blockchain based on PHR.	Lack of legal and institutional mechanisms that can support activation of blockchain.
[54]	Storing EHRs, clinical trial reports and creating machine learning models based on this data	Insecure storage of highly private and critical data	--	Depending upon the needs, various unsupervised and supervised algorithms can be implemented	Standard tamper-proof append only ledger, much like the cryptocurrency Bitcoin’s Ledger	---	Developing a secure and trusted data management platform	Efficiency of the models will depend on the quality of data stored in the blockchain and the degree of tamper resilience will depend on how well the blockchain has been implemented.
[55]	Quick and efficient storage, retrieval, and sharing of electronic medical records and smart model for updated report generation	Insecure transfer of data	The data uploaded by the patients through the network is used as the dataset for the training purposes.	Natural language processing, Convolutional Neural Networks, Image Processing, and Encoding-Decoding.	No particular type has been mentioned in the text.	Ethereum-Based Blockchain	Smart electronic medical report handling and Usage	Bugged Ethereum contracts can render the system highly inefficient.
[56]	Developing a voting-based smart diagnosis system where data can be stored and shared on a blockchain among various health centres.	--	The data present in the blockchain will serve as the training data for the models present with each healthcare centre	Artificial Neural Networks, Support vector machines, Decision trees, Random Forests, AdaBoost, and Bayesian Networks	No particular type has been mentioned in the text	No particular type has been mentioned in the text	Smart voting-based disease diagnosis system	This system will be as efficient as the quality of data present on the blockchain.
[57]	Handling patient data, health records, AI-powered Diagnosis, optimized testing, Radiological and Psychological deductions, smart drug delivery	Privacy and Anonymity, Usability of data, and consent of patients and organizations.	Available data on the blockchain serves as the datasets	Random Forest Classifiers and Regression, Deep learning algorithms such as Convolutional Networks and Recurrent Neural Networks.	No particular type has been mentioned in the text	No particular type has been mentioned in the text	Different spheres of Medical Diagnostics and data management that can benefit from the combination of machine learning and Blockchain technologies.	---
[58]	Using machine learning and blockchain technology for Cancer care	--	Combination of data present in different institutions and organizations connected by blockchain along with environment-based factors and regional healthcare service assessment of the survivors	No particular algorithms have been named in this work	No particular type has been mentioned in the text	No particular type has been mentioned in the text	Conceptualized a system that can detect cancer recurrence and prognosis using machine learning	This system will be as efficient as the quality of data present on the blockchain
[59]	Sharing personal continuous-dynamic health data.	Centralised data storage methods hinder sharing and has single point of attack	--	Conceptual usage of advanced machine learning algorithms.	Public blockchain type	Ethereum, Hyperledger Fabric	Data sharing and transaction validation along with quality validation module using machine learning.	Security issues once the data are transferred to the customer are not discussed.

**Table 4:** A summary of works on deep learning-enabled Blockchain Technologies for Public Health.

Ref.	Healthcare Application	Security Challenges	Dataset	Deep Learning Model Used	Blockchain Type Used	Blockchain Platform	Key Contribution	Limitations
[61]	Blockchain-deep learning environment for analyzing EHRs.	EHR needs to be secured against unauthorized access and cyber-attacks.	EHR data from some hospitals are used after permission.	RNN-Long Short-Term Memory (RNN-LSTM).	Permissioned blockchain network/private blockchain.	Hyperledger.	Store EHR of patients using Hyperledger and analyze blocks using RNN-LSTM and RNN-GRU.	High maintenance coset compared to traditional models.
[59]	Detection of myopia	Data transfer and sharing between collaborators for medical studies.	Retinal photographs from Singapore, China, Taiwan, and India.	A new model developed to detect myopia.	Permissioned blockchain network/private blockchain.	Hyperledger.	Detecting internal diseases such as Myopia.	The severity of the problem is not identified.
[69]	Secure way of sharing EHRs amongst healthcare users.	Threat to availability and integrity of the EHRs. Collusion attacks are also possible in the given scenario.	EHR records from different hospitals.	A deep learning model consisting of 4 layers is suggested.	In the suggested blockchain model, n lattices-based cryptography has been used.	Post-quantum blockchain networks are used.	A blockchain-based deep learning as-a-service model is suggested which ensures secure and accurate sharing of EHRs.	Scalability and complexity of the proposed model.
[70]	To carry out accurate DNA diagnosis of Malaria and blockchain technology is used for secure management of the dataset.	Secure data connectivity between different geo-locations in rural areas is quite difficult and the management of the dataset is also essential.	Dataset was taken from field tests in rural Uganda.	--	--	Hyperledger Composer.	--	--
[71]	Blockchain convolution neural networks and audio-video emotion patterns to detect healthcare emergencies in nearby areas.	Providing security to patients’ reports and other medical services is quite essential and there can be a major threat of external parties accessing the patient’s details.	Audio-visual patterns will help in training the model of emotional recognition.	CNN deep learning techniques are used.	Blockchain convolution neural networks.	Ethereum ecosystem has been used.	Contributed to the field of emotional recognitions using deep learning and it provides a layer of security as well using smart contracts.	Not included recurrent network methods which can help to predict results for an existing or past patient.
[72]	Suggested a secure model with the application of blockchain framework Exonum and deep learning algorithms to manage control over personal data in medical records.	The data possessed by patients in form of their medical records hold great value for predictive analysis and securing that data, which is limiting access to the patient and doctor, it possesses a major challenge.	The dataset of some patients has been taken with their consent to apply different deep learning and blockchain algorithms.	GAN-generative adversarial networks.	Exonum framework blockchain.	Exonum.	Suggested a system that can reduce the difficulty of carrying out biomedical research.	Limited to text-form of data and other forms have not been considered in the database.
[73]	The proposed model is formed to identify feature-extracted data from the existing data. Deep Neural networks and blockchain smart contracts have been applied to the model.	EHRs have a centralized database and that is a major security issue since the control belongs to a single person.	Greater Noida COVID-19 dataset is used.	The DNN model is trained for feature extraction. The major diseases in a particular area.	Blockchain with a token-based approach is used.	Ethereum virtual machine.	The bulk data are reduced to data size which can simply be predictive analysis.	More advanced versions of blockchain could have been used.

## Data Availability

Not applicable.
